# Genomic analyses of complex *P. vivax* infections

**DOI:** 10.1186/1475-2875-13-S1-O41

**Published:** 2014-09-22

**Authors:** Ernest Chan, Didier Menard, John Barnwell, Peter Zimmerman, David Serre

**Affiliations:** 1 Cleveland Clinic, Cleveland, Ohio, USA; 2 Institut Pasteur du Cambodge, Phnom Penh, Cambodia; 3 Centers for Disease Control and Prevention, Atlanta, Georgia, USA; 4 Case Western Reserve University, Cleveland, Ohio, USA

## Background

The occurrence of multiple, genetically different, parasites in *P. vivax* -infected patients is well known but often conveniently disregarded. This complexity of infection has long been difficult tostudy due to a lack of molecular markers. Genome sequencing approaches could circumvent this limitation but require tailored analyses that have not always been implemented.

## Materials and methods

Here, we analyze genome sequence data generated by us and others from *P. vivax* field isolates and monkey-adapted strains to characterize, across the entire genome, SNPs and sequence rearrangements. We also genotyped several blood samples infected by the same monkey-adapted strain to better understand the changes happening during the generation of these strains.

## Results

All field isolates sequenced so far show evidence of complex infections, with typically 2-4 strains present at >5%. We show that it is possible, empirically and analytically, to rigorously differentiate these parasites and reconstruct haploid genome sequences from these complex infections. Our analyses also demonstrate that the presence of distinct parasites in the original patient infection can lead to genetic heterogeneity in monkey-adapted strains.

## Conclusions

Our findings reveal the pervasiveness of complex infections in *P. vivax* and show that erroneous genetic conclusions can be made if this aspect of the parasite’s biology is not carefully addressed. We show that different approaches enable to rigorously assess complexity of infection and to separate strains within an infection. In addition to their relevance for genetic and genomic studies, our findings have important implications for *in vitro* and *ex vivo* studies of *P. vivax* and provide a framework to better design and control such functional studies.

